# MicroRNA-126 selected with broad-spectrum analysis of microRNAs – a new predictive factor for the effectiveness of immunotherapy or chemoimmunotherapy in advanced NSCLC patients?

**DOI:** 10.3389/fimmu.2024.1344858

**Published:** 2024-02-26

**Authors:** Anna Grenda, Barbara Kuźnar-Kamińska, Ewa Kalinka, Paweł Krawczyk, Marek Sawicki, Agata Filip, Izabela Chmielewska, Małgorzata Frąk, Natalia Krzyżanowska, Janusz Milanowski

**Affiliations:** ^1^ Department of Pneumonology, Oncology and Allergology, Medical University of Lublin, Lublin, Poland; ^2^ Department of Pulmonology, Allergology and Pulmonary Oncology, Poznan University of Medical Sciences, Poznań, Poland; ^3^ Department of Oncology, Polish Mother’s Memorial Hospital Research Institute, Łódź, Poland; ^4^ Department of Thoracic Surgery, Medical University of Lublin, Lublin, Poland; ^5^ Department of Cancer Genetics with Department of Cancer Genetics with Cytogenetics Laboratory, Medical University in Lublin, Lublin, Poland

**Keywords:** microRNA, immunotherapy, anti-PD-1, NSCLC, miRNA

## Abstract

**Introduction:**

Expression of PD-L1 on cancer cells is the only validated predictive factor for immunotherapy in NSCLC (Non-Small Cell Lung Cancer) patients. However, on this basis, it is difficult to predict the occurrence of resistance to immune checkpoint inhibitors (ICIs). MicroRNAs are widely studied as biomarkers of cancers. Our study was designed to determine whether microRNAs can be sensitive predictive factors in the qualification of NSCLC patients to first-line immunotherapy or chemoimmunotherapy.

**Material and methods:**

The two-stage research on validation group (n=20) and study group (n=35) of patients with advanced NSCLC was conducted. Analysis of microRNAs expression by qPCR in plasma collected prior to the start of immunotherapy (pembrolizumab) or chemoimmunotherapy (combination of pembrolizumab with chemotherapy) was made. Broad-spectrum analysis of microRNAs expression was used in the studied group. Three microRNAs selected in that group as important for the effectiveness of ICIs were then examined in the validation group.

**Results:**

In the studied group, significantly higher expression of miRNA-126-3p, miR-144-3p and miR-146-5p was observed in patients with long PFS compared to those with short PFS. In the validation group, low miRNA-126 expression indicated lower median progression-free survival and overall survival (2.3 vs. 5.0 months and 5.2 vs 11.2, respectively). These patients had a significantly higher risk of progression (HR= 2.92, 95% CI: 1.01 to 8.40, p=0.04) and death (HR=3.64, 95% CI: 1.22 to 10.84, p=0.02).

**Conclusion:**

Our study showed that the expression of miR-126 in blood plasma may be a predictive factor for the effectiveness of first-line immunotherapy or chemoimmunotherapy in advanced NSCLC patients.

## Introduction

The percentage of tumor cells (TC) with PD-L1 (Programmed Cell Death Ligand 1) expression is determined during the qualification of patients with advanced non-small cell lung cancer (NSCLC) to therapy with immune checkpoint inhibitors (ICIs) in monotherapy or combination with chemotherapy. Atezolizumab, pembrolizumab or cemiplimab can be used in first-line monotherapy when PD-L1 expression is observed on ≥50% of TC. Immunotherapy combined with chemotherapy can be considered for first-line therapy if PD-L1 expression is found on less than 50% of tumor cells. For advanced NSCLC patients with PD-L1 expression on less than 1% of TC or regardless of this expression, pembrolizumab or nivolumab or atezolizumab could be used in second-line therapy in patients who have not previously received immunotherapy (1–14).

PD-L1 is the only validated predictor of immunotherapy efficacy, but it is not perfect. The probability of disease progression and resistance to ICIs therapy cannot be accurately determined based on PD-L1 expression. Approximately 40% of patients with high PD-L1 expression have primary resistance to immunotherapy and show disease progression. Another 30% of patients achieve disease stabilization with a short progression-free survival (PFS) of approximately 6 months. These patients develop an acquired resistance to immunotherapy. However, in the remaining patients, the response to immunotherapy is long-lasting (11–13). Moreover, immunotherapy may be highly beneficial in patients with low or no expression of PD-L1 protein on TCs. The mechanism of resistance to immunotherapies is not fully understood (1, 14). Resistance to immunotherapy may be influenced by intrinsic factors of the cancer cell, such as epigenetic factors and disruption in gene expression, as well as mutations that compose the molecular landscape of the cancer cells (15–17). The action of immunosuppressive cytokines or growth factors, neoangiogenesis associated with the formation of abnormal blood vessels, expression of molecules on T cells that send signals to silence the immune system, tumor-infiltrating lymphocyte (TIL) status, or the composition of the gut microbiome belong to the external factors, independent of the tumor cells (17, 18).

Our attention was drawn to epigenetic factors which are microRNA molecules. They are short in length (~21nt), stable, and present in plasma/serum, which ensures easy availability of material for testing, without the need for invasive methods. MicroRNAs affect almost all cellular processes, by regulating gene expression at the post-transcriptional level. Expression of microRNAs changes under pathological conditions, including cancer development. Thus, they may be related to all molecular and immunological mechanisms of resistance to immunotherapy. MicroRNAs are widely studied as precise biomarkers in the context of early cancer diagnosis. Our present study was designed to determine whether they can be sensitive predictive factors for the effectiveness of immunotherapy or chemoimmunotherapy in patients with advanced NSCLC.

## Materials and methods

### Patients characteristic

The study consisted of two stages. The first involved the selection of microRNAs from a panel of miRCURY LNA Human Serum/Plasma Focus PCR Panels (Qiagen, Venlo, Netherlands) and was conducted on a group of 20 NSCLC patients (in stage IV) treated with immunotherapy (10 patients with PD-L1 expression on ≥50% of TC) or chemoimmunotherapy (10 patients with PD-L1 expression on <50% of TC).

Patients were divided based on the length of progression-free survival. Ten patients were characterized by short disease stabilization or disease progression with a PFS of less than 6 months and 10 patients had a PFS longer than 6 months. There were 6 (30%) women and 14 (70%) men. 13 (65%) patients were over 65 years of age and 7 (35%) patients were under 65 years of age. All patients were in stage IV according to 8^th^ TNM classification. There were 7 patients diagnosed with squamous cell carcinoma and 13 patients with adenocarcinoma.

In the second stage, we performed assays on an independent, validation group of patients with selected micoRNAs (from the first stage of the study) that were classified as potential predictors of immunotherapy or chemoimmunotherapy efficacy. The group consisted of 35 patients. Thirty-four patients were in stage IV and one patient in stage IIIB of the disease. *EGFR* mutations (*Epidermal Growth Factor Receptor*), *ALK* (*Anaplastic Lymphoma Kinase Tyrosine Kinase Receptor*) and *ROS1* (*ROS Proto-Oncogene 1, Tyrosine Kinase Receptor*) rearrangement were excluded in all patients. Responses to immunotherapy, progression-free survival, and overall survival were calculated from the start of therapy in all 35 patients.

Demographic and clinical characteristics of the entire study group are presented in **Table 1**.

**Table 1:** Clinical and demographic characteristics of patients in the validation group (n=35).

Characteristic	miRNA-126 n (%)		miRNA-144 n (%)		miRNA-146 n (%)	
	Below the median	Above the median	Below the median	Above the median	Below the median	Above the median
**Age (median=69 years, min-max: 48-77, SD=6.4** **<69 n=13** **≥69 n=22**	1 (8) 8 (36)	12 (92) 14 (64)	5 (38) 12 (55)	8 (62) 10 (45)	4 (31) 12 (55)	9 (69) 10 (45)
***X* ^2^** ***p***	3.51 0.06		0.84 0.36		1.86 0.17	
**Gender** Male n=20 Female n=15	6 (30) 3 (20)	14 (70) 12 (80)	10 (50) 7 (47)	10 (50) 8 (53)	9 (45) 7 (47)	11 (55) 8 (53)
***X^2^*** ***p***	0.45 0.50		0.04 0.85		0.01 0.92	
**Histopathology** Non-SqC, n=24 SqC n=11	8 (33) 1 (9)	16 (67) 10 (91)	11 (46) 6 (55)	13 (54) 5 (45)	10 (42) 6 (55)	14 (58) 5 (45)
***X^2^*** ***p***	2.32 0.13		0.23 0.63		0.50 0.48	
**PD-L1 IHC** <50% n=12 ≥50% n=23	4 (33) 5 (22)	8 (67) 18 (78)	6 (50) 11 (48)	6 (50) 12 (52)	7 (58) 9 (39)	5 (42) 14 (61)
***X^2^*** ***p***	0.56 0.46		0.015 0.90		1.17 0.28	
**Response to immunotherapy** PD n=14 SD+ PR n=21	6 (43) 3 (14)	8 (57) 18 (86)	8 (57) 9 (43)	6 (43) 12 (57)	6 (43) 10 (48)	8 (57) 11 (52)
***X^2^*** ***p***	3.59 0.06		0.68 0.41		0.078 0.78	
**PFS** <6onths n=25 ≥6months n=10	8 (32) 1 (10)	17 (68) 9 (90)	14 (56) 3 (70)	11 (44) 7 (70)	14 (56) 2 (20)	11 (44) 8 (80)
***X^2^*** ***p***	1.81 0.18		1.93 0.16		3.73 0.05	
**OS** <6onths n=19 ≥6months n=16	6 (32) 3 (19)	13 (68) 13 (81)	11 (58) 6 (37)	8 (42) 10 (63)	9 (47) 7 (44)	10 (53) 9 (56)
***X^2^*** ***p***	0.74 0.39		1.45 0.23		0.05 0.83	

The research was approved by the bioethics committee at the Medical University of Lublin (KE-0254/95/2018). Informed consent was obtained from all patients.

### Sample collection

The material for the study consisted of plasma samples taken from patients before the treatment. The blood was collected in EDTA (ethylenediaminetetraacetic acid) tubes and centrifuged for 10 min at 2000 x g. The plasma was pipetted in equal amounts into eppendorf tubes. Plasma samples were stored at -80°C until isolation of RNA was carried out.

### MicroRNA expression testing in the experimental group

Isolation of free-circulating microRNAs was performed using the miRNeasy Serum/Plasma Kit (Qiagen, Venlo, Netherlands). The isolated RNA was stored at -80°C until the reverse transcription reaction was performed. Reverse transcription reactions were performed using miRCURY^®^ LNA^®^ RT Kit (Qiagen, Venlo, Netherlands) according to the manufacturer’s instructions on the T Personal instrument (Analitik Jena, Jena, Germany). MicroRNAs expression was evaluated by qPCR in 20 patients from the experimental group using the miRCURY LNA Human Serum/Plasma Focus PCR Panels (Qiagen, Venlo, Netherlands) kit on the Applied Biosystems 7500 Fast Real-Time PCR System (Applied Biosystems, Waltham, USA). The 10-microliter reaction was prepared according to the manufacturer’s instructions. qPCR was performed according to the following time and temperature conditions: PCR initial heat activation: 2 min, 95°C, and next 40 cycles: denaturation 10 s, 95°C, annealing/extension 60 s, 56°C. A melting curve analysis was attached to each run. The obtained Ct values were used for calculations using the method 2^-ΔCt^. MiRNA-484 and cel-miR-39-3p spike-in were used as controls.

### MicroRNA expression testing in the validation group

Isolation of free-circulating miRNAs was performed using miRNeasy Serum/Plasma Kit (Qiagen, Venlo, Netherlands). The isolated RNA was stored at -80°C until the reverse transcription reaction was performed. For cDNA synthesis TaqMan™ Advanced miRNA cDNA Synthesis Kit (ThermoFisher Scientific, Waltham, Massachusetts, USA) was used.

Three microRNAs were selected for further validation analyses among the microRNAs tested in the experimental group. These microRNAs showed significantly different expression in patients with short and long PFS in the experimental group. The tests were performed using TaqMan probes. Expression of miRNA-126-3p (cat. A25576 477887_mir), miR-144-3p (cat. A25576 477913_mir), miR-146a-5p (cat. A25576 478399_mir) were examined. Expression of miRNA-484 (A25576 478308_mir) and cel-miR-39-3p (cat. A25576 478293_mir) spike-in were used as controls. Reactions were performed on the illumina Eco Real-Time PCR system. The 20 microliter reaction contained: 10 μl TaqMan™ Fast Advanced Master Mix (ThermoFisher Scientific, Waltham, Massachusetts, USA), 1 μl TaqMan^®^ Advanced miRNA Assay, 4 μl nuclease free water and 5 µL of the diluted cDNA (1:10) template. Temperature conditions were used as follows: enzyme activation 95°C by 20 seconds and then 40 cycles: denature 95°C by 3 seconds and anneal/extend 62°C by 30 seconds. The obtained Ct values were used for calculations using the 2^-ΔCt^ method.

### Statistical analysis

Statistical analysis was performed using Statistica 13.3 (TIBCO Software Inc, Palo Alto, USA) and MedCalc (MedCalc Software Ltd, Ostend Belgium) software. The U-Mann–Whitney test was used to assess differences in miRNA expression between groups of patients. Kaplan-Meier survival analysis were used for the calculation of PFS and OS. The results are presented as medians and maximum and minimum values (min-max). A p-value below 0.05 was considered statistically significant.

## Results

The median PFS in the experimental group was 1.9 months (95%CI: 1.5 to 11.6, min-max: 1.0-23.6). The median overall survival calculated from the start of immunotherapy was 6.6 months (95%CI: 1.7 to 13.8, min-max: 1.0-23.6).

**Figure 1:**
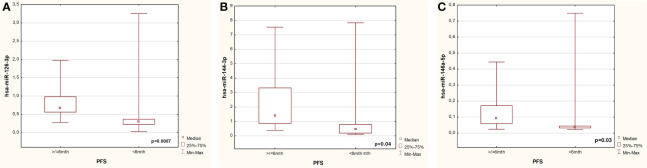
Differences in expression of miRNA-126 **(A)**, miR-144 **(B)**, miR-146 **(C)** in patients with short and long PFS from the experimental group.

Based on the analysis of microRNAs expression in experimental group, significantly higher expression of miRNA-126-3p (p=0.007), miR-144-3p (p=0.04) and miR-146-5p (p=0.03) was observed in patients with long PFS compared to those with short PFS (**Figures 1A–C** respectively).

**Figure 2:**
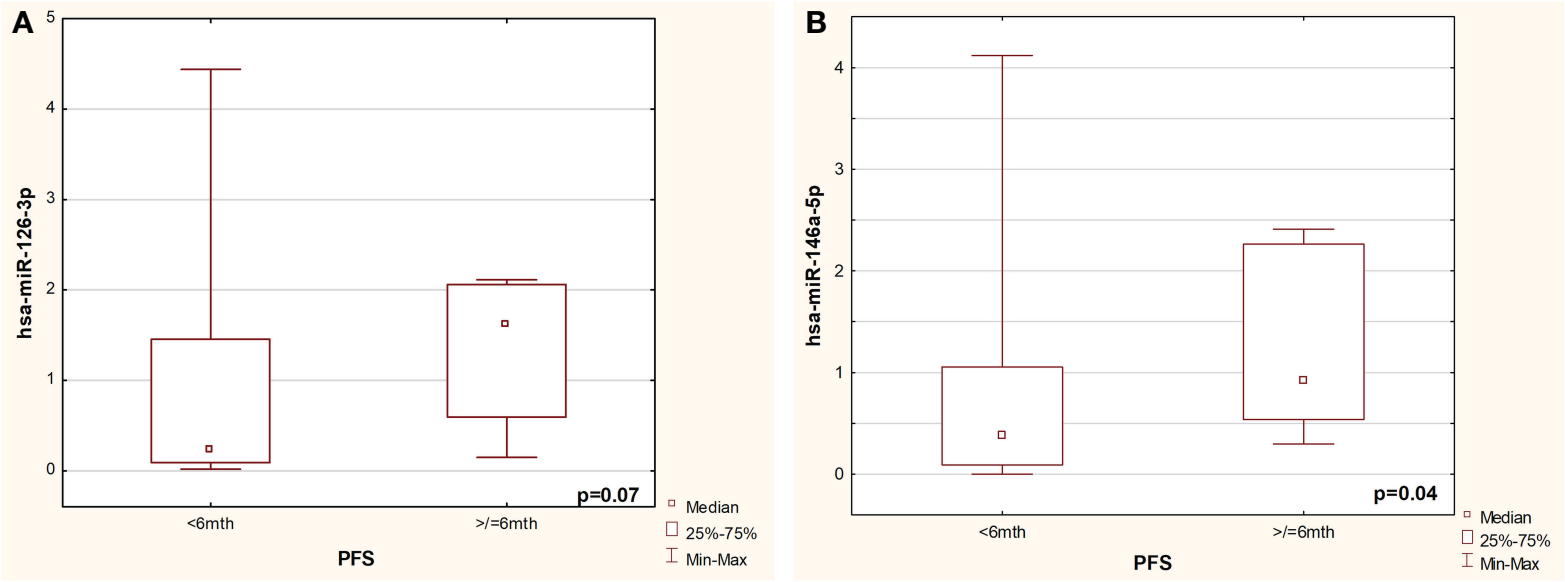
Comparison of expression of miRNA-126-3p **(A)** and miR-146-5p **(B)** in patients with short and long PFS from validated group.

Therefore, further studies in the validation group focused on these three microRNA molecules. In an independent validation group, miRNA-126 expression was non-significantly higher in patients with PFS over 6 months (p=0.07, **Figure 2A**) in comparison to patients with shorter PFS. Significantly higher expression of miRNA-146 (p=0.04, **Figure 2B**) was found in patients with long PFS than in patients with short PFS. No such differences were observed during analysis of the miRNA-144 expression in these groups (p=0.5).

**Figure 3:**
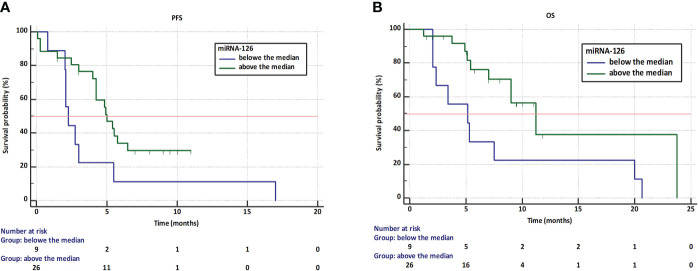
Kaplan-Meier curves showing progression-free survival **(A)** and overall survival **(B)** in NSCLC patients treated with first-line immunotherapy or chemoimmunotherapy with different expression of miRNA-126-3p.

Kaplan-Meier analysis showed that the median PFS was lower in patients with low expression of miRNA-126 compared to patients with high expression of this molecule (2.3 vs. 5.0 months). The risk of progression was almost three times higher in the group of patients with low expression of the tested microRNA compared to patients with high expression of this molecule (HR= 2.92, 95% CI: 1.01 to 8.40, p=0.04, **Figure 3A**). Moreover, median OS was lower in patients with lower miRNA-126 expression than in patients with higher miRNA-126 expression (5.2 vs. 11.2 months). Lower expression of miRNA-126 indicated almost four times higher risk of OS shortening (HR=3.64, 95% CI: 1.22 to 10.84, p=0.02, **Figure 3B**).

## Discussion

We selected three microRNAs which expression could be a predictive factor for the efficacy of immunotherapy with anti-PD-1 antibodies. For this purpose, we used an analysis of a broad panel of miRNA molecules in the plasma of NSCLC patients treated with first-line immunotherapy or chemoimmunotherapy. Expression of miRNA-126-3p, miRNA-144-3p and miRNA-146a-5p have been indicated as potentially useful factors in the qualification for immunotherapy. In further studies in an independent validation group, we found that high miRNA-126-3p expression could be a predictive factor for first-line immunotherapy efficacy in non-small cell lung cancer patients. It should be mentioned that the expression of miRNAs, including miRNA-126, may be influenced by chemotherapy or other method of treatment. Our goal was to evaluate whether any miRNA molecules expression could be a universal biomarker of response to immune checkpoints inhibitors used alone or in combination with chemotherapy in advanced NSCLC patients. Therefore, we analyzed these potential predictive factors before starting treatment.

Other authors’ research indicated that miRNA-126 expression is significantly reduced in adenocarcinoma of the lung compared to the normal tissue. Therefore, reduction of miRNA-126 expression is associated with the development of lung adenocarcinoma (LUAD) (19). Moreover, lower expression of miRNA-126-3p and miRNA126-5p promotes vascular invasion, and lymph node metastasis, and occurs in higher stages (III-IV) of adenocarcinoma patients.

The target transcripts for miRNA-126 activity were identified: *IGF2BP1* (*Insulin Like Growth Factor 2 MRNA Binding Protein 1*), *TRPM8* (*Transient Receptor Potential Cation Channel Subfamily M Member 8*), *DUSP4* (*Dual Specificity Phosphatase 4*), *SOX11* (*SRY-Box Transcription Factor 11*), *PLOD2* (*Procollagen-Lysine,2-Oxoglutarate 5-Dioxygenase 2*), *LIN28A* (*Lin-28 Homolog A*), *LIN28B* (*Lin-28 Homolog B*), *SLC7A11* (*Solute Carrier Family 7 Member 11*), *mTOR* (*Mechanistic Target Of Rapamycin Kinase*)*, PIK3R2* (*Phosphoinositide-3-Kinase Regulatory Subunit 2*) (19–21). MiRNA-126 has tumor suppressor properties. MiRNA-126 has an inhibitory effect on NSCLC cell invasion by silencing oncogenes: *VEGFA* (*Vascular Endothelial Growth Factor A), AKT1 (AKT Serine/Threonine Kinase 1*) and *KRAS* (*Kirsten Rat Sarcoma Virus Proto-Oncogene, GTPase*) (22). Moreover, it has been found that miR-126-3p inhibits the growth, migration, and invasion of NSCLC by targeting *CCR1* (*C-C Motif Chemokine Receptor 1*) in NSCLC cells (23). However, it was demonstrated that the over-expression of CCR1 molecule rescued the inhibitory effects of miR-126-3p on NSCLC cell growth, migration and invasion. Further, the knocked-down of CCR1 was able to mimic the inhibitory effects of miR-126-3p on the progression of NSCLC cells (23).

Di Paolo et al. identified in a group of 38 NSCLC patients that the expression of miR-126-3p and miR-221-3p was significantly changed in tumor tissue compared to healthy tissue (24). They found that concomitant miR-126-3p activation and miR-221-3p inhibition reduced lung cancer cell viability by inhibiting AKT, PIK3R2 and PTEN (Phosphatase And Tensin Homolog) signaling pathways (24). *PIK3R2* was a target for the action of miR-126-3p and *PTEN* for miR-221-3p. Researchers proved that the simultaneous interaction of these molecules reduced metastatic dissemination of lung cancer cells both *in vitro* and *in vivo* through CXCR4 (C-X-C Motif Chemokine Receptor 4) inhibition. Further, Ichikawa et al. showed miR−126−3p could inhibit cell migration and invasion and induce apoptosis by regulating the PI3K/PDK1/AKT pathway in HeLa cells (25). A meta-analysis of Sun et al. showed that generally high expression of miR-126 is associated with better prognosis in NSCLC patients (26). In research by Yang et al., expression of miRNA-126 was decreased in NSCLC lines and tumor tissues. The patients with low expression of miRNA-126 had significantly poorer overall survival than those with high miRNA-126 expression (means OS reached 24.4 vs. 29.3 months, respectively) (27).

It has also been shown that miRNA-126-3p down-regulation contributes to dabrafenib-acquired resistance in melanoma patients by up-regulating *ADAM9* (*ADAM Metallopeptidase Domain 9*) and *VEGFA* (28). This is consistent with the observation that decreased *ADAM9* mRNA expression correlated with a better response to nivolumab therapy in hepatocellular cancer (29). Liu et al. reported that miR-126 suppressed esophageal cancer cell proliferation and migration by interacting with *ADAM9* mRNA 3′UTR (Untranslated Region) (30). They indicated that expression of miR-126 was reduced in esophageal cancer tissues, which was correlated with shorter overall survival of patients, implying their potential function as a prognostic factor (30). In contrast, in colorectal cancer, high expression of miR-126 in tumor and stroma was associated with increased overall survival. In multivariate analyses, high miR-126 expression in tumor remained a significant independent predictor of improved survival (31). The authors postulate that this factor may help in the qualification of patients for adjuvant chemotherapy (31).

In turn, Schmittnaegel et al. postulated that the blockade of Ang2 (angiopoietin-2) and VEGFA induces antitumor immunity enhanced by PD-1 checkpoint blockade (32). These are indications that the expression of the miRNA-126-3p molecule is a beneficial factor for NSCLC patients receiving immunotherapy. Researchers indicated that the miR-126 molecule is associated with the functioning of T lymphocytes, especially Treg cells. Chen et al. postulated that investigation of the role of miR-126 in lung cancer development and progression, including in activation of the immune response, may be valuable for the estimation of immunotherapy efficacy (22).

The previous considerations and our results indicate that higher miR-126 expression may be a favorable predictive factor for immunotherapy. Qin et al. found that miRNA-126 was expressed in mouse and human Treg cells (33). It has been shown that miRNA-126 regulates the activity of the PI3K-AKT signaling pathway, crucial for Foxp3 (Forkhead Box P3) expression, and limited activation of PI3K/AKT pathway was necessary for Tregs development and function. Researchers in further studies showed that silencing of miRNA-126 using antisense oligonucleotides (ASO) could significantly reduce the induction of Treg cells *in vitro*. Furthermore, miR-126 silencing could reduce the expression of Foxp3 on Treg cells, which was accompanied by decreased expression of CTLA-4 (Cytotoxic T-Lymphocyte Associated Protein 4) and GITR (TNF Receptor Superfamily Member 18), as well as IL-10 and TGF-β production. Therefore, high expression of miRNA-126 in lymphocytes may be associated with the activation, differentiation and suppressive activity of Treg cells. Moreover, Fortunato et al. found that high level of miRNA-126-3p in plasma may be related to the induction and activation of Treg cells, which enhance the metabolism and secretion of exosomes containing microRNAs, including miRNA-126-3p (34). In turn, high activity of Treg cells in the tumor microenvironment and lymph nodes is associated with immunosuppression and poorer effectiveness of immunotherapy. However, the function of Treg lymphocytes is only one of many immunological factors influencing the efficacy of immunotherapy. One of them may be the function of monocytes on the spreading of cancer. Zhang et al. indicated that miRNA-126 independently suppress the sequential recruitment of mesenchymal stem cells and inflammatory monocytes into the tumor stroma. The lack of these cells in the microenvironment may be favorable for metastases development in the breast cancer mouse xenograft model (35).

## Conclusion

Our study shows that miR-126 may be a predictive factor of the effectiveness of first-line immunotherapy or chemoimmunotherapy in NSCLC patients. We are aware that our study groups were not very large. We postulate that further research should be carried out in a larger group and in patients treated with second-line immunotherapy to investigate whether the expression of this molecule has predictive properties in these patients.

## Data availability statement

The raw data supporting the conclusions of this article will be made available by the authors, without undue reservation.

## Ethics statement

The studies involving humans were approved by Bioethics Committee at the Medical University of Lublin. The studies were conducted in accordance with the local legislation and institutional requirements. The participants provided their written informed consent to participate in this study.

## Author contributions

AG: Conceptualization, Formal analysis, Methodology, Project administration, Writing – original draft. BK: Conceptualization, Formal analysis, Investigation, Resources, Writing – original draft. EK: Data curation, Formal analysis, Methodology, Writing – review & editing. PK: Conceptualization, Supervision, Writing – original draft, Writing – review & editing. MS: Data curation, Investigation, Supervision, Writing – review & editing. AF: Software, Supervision, Writing – review & editing. IC: Data curation, Formal analysis, Investigation, Resources, Visualization, Writing – original draft. MF: Data curation, Formal analysis, Writing – original draft. NK: Data curation, Writing – review & editing. JM: Supervision, Writing – review & editing.
